# The Effect of Acceptor and Donor Doping on Oxygen Vacancy Concentrations in Lead Zirconate Titanate (PZT)

**DOI:** 10.3390/ma9110945

**Published:** 2016-11-22

**Authors:** Christoph Slouka, Theresa Kainz, Edvinas Navickas, Gregor Walch, Herbert Hutter, Klaus Reichmann, Jürgen Fleig

**Affiliations:** 1 Institute of Chemical Technologies and Analytics, Vienna University of Technology, Getreidemarkt 9/164EC, 1060 Vienna, Austria; christoph.slouka@tuwien.ac.at (C.S.); edvinas.navickas@tuwien.ac.at (E.N.); gregor.walch@tuwien.ac.at (G.W.); h.hutter@tuwien.ac.at (H.H.); 2 Institute for Chemistry and Technology of Materials, Graz University of Technology, Stremayrgasse 9, 8010 Graz, Austria; theresa.kainz@epcos.com (T.K.); k.reichmann@tugraz.at (K.R.)

**Keywords:** defect chemistry, oxygen vacancies, doping, diffusion, lead zirconate titanate

## Abstract

The different properties of acceptor-doped (hard) and donor-doped (soft) lead zirconate titanate (PZT) ceramics are often attributed to different amounts of oxygen vacancies introduced by the dopant. Acceptor doping is believed to cause high oxygen vacancy concentrations, while donors are expected to strongly suppress their amount. In this study, La^3+^ donor-doped, Fe^3+^ acceptor-doped and La^3+^/Fe^3+^-co-doped PZT samples were investigated by oxygen tracer exchange and electrochemical impedance spectroscopy in order to analyse the effect of doping on oxygen vacancy concentrations. Relative changes in the tracer diffusion coefficients for different doping and quantitative relations between defect concentrations allowed estimates of oxygen vacancy concentrations. Donor doping does not completely suppress the formation of oxygen vacancies; rather, it concentrates them in the grain boundary region. Acceptor doping enhances the amount of oxygen vacancies but estimates suggest that bulk concentrations are still in the ppm range, even for 1% acceptor doping. Trapped holes might thus considerably contribute to the charge balancing of the acceptor dopants. This could also be of relevance in understanding the properties of hard and soft PZT.

## 1. Introduction

The ferroelectric and piezoelectric properties of perovskite-type titanates are employed in many technological devices such as positive temperature coefficient (PTC) resistors and multilayer capacitors [1], generators, motors, ultrasonic transductors, actuators, capacitors, or non-volatile memories [2,3]. Knowledge of the ionic and electronic conductivity in these oxides is highly relevant for establishing, optimizing, and retaining their functionality. For barium titanate and strontium titanate, many studies are available dealing with defect concentrations and transport properties [4,5,6,7,8,9,10,11,12,13,14]. In lead zirconate titanate (PZT), defect-related properties such as the mixed ionic and electronic conductivity are much less understood. This lack of knowledge is partly caused by the limited control of cation stoichiometry due to PbO losses during sintering. 

Still, doping is often used to tailor properties of PZT. So-called hard PZT ceramics mainly result from acceptor doping, e.g., by Fe^3+^, and these ceramics exhibit less sharp hysteresis loops and lower dielectric constants. Their behaviour, such as difficult poling but also high mechanical quality factor, is often attributed to the supposedly high concentration of oxygen vacancies and their interaction with domain walls. Early electron paramagnetic resonance (EPR) spectra of Fe in PbTiO_3_ gave hints for two different iron centres, one of them being interpreted as a defect associate of Fe^3+^ with its compensating oxygen vacancy [15]. This was later confirmed [16,17], again with EPR spectra. Recently, further details on point defect associates and their interaction with domain walls were revealed by density functional theory calculations [18,19] and analysis of hysteresis loops [20]. Also trapping of oxygen vacancies in crystallographic shear planes was reported [21].

Soft PZT ceramics result, for example, by doping with Nb^5+^ and exhibit properties such as square hysteresis loops, low coercive fields, high remnant polarization, high dielectric constants, maximum coupling factors, higher dielectric loss, high mechanical compliance, and reduced aging. Essential in this context is the fact that mainly immobile cation vacancies result from donor doping. For details on the current status of the interpretation of hardening and softening of PZT, see [18,22,23].

The effects of donor doping were already summarised in 1971 in [24]. A more comprehensive study on rare earth doping can be found in [25]. It was also shown that donor doping does not lead to complete annihilation of the p-type conduction behaviour in PZT and furthermore does not entirely suppress the formation of oxygen vacancies [26,27,28,29,30,31]. Oxide ion motion was analysed for undoped and donor doped PZT as well as co-doped PZT with donor excess [32,33,34,35,36]. Among others, an enhanced oxide ion conductivity along grain boundaries was found in nominally donor-doped PZT [35,37,38]. Still, quantitative data on the defect chemical properties of doped and co-doped PZT are scarce. Additional studies on the charge balancing defects in doped PZT are therefore highly desirable in order to further elucidate the reasons behind hard and soft piezoelectric behaviour.

In this contribution, we discuss the transport properties of donor-doped PZT, acceptor-doped PZT, and combined acceptor–donor-doped PZT with acceptor excess. Defect chemical information is gained from a combined study using tracer diffusion, analysed by subsequent time-of-flight secondary ion mass spectrometry (ToF-SIMS) and electrochemical impedance spectroscopy (EIS). Oxygen tracer diffusion coefficients scale with the oxygen vacancy concentration and thus comparison between samples reveals relative changes of the vacancy concentration caused by doping. EIS and the partial pressure dependence of the conductivity allow the analysis of the conducting species and relative concentration changes of the electronic charge carriers with doping. Applying this combination of techniques to sample series of La^3+^ donor-doped, Fe^3+^ acceptor-doped, and Fe/La co-doped PZT allowed us to draw conclusions on oxygen vacancy concentrations in differently doped PZT.

## 2. Experimental

### 2.1. Sample Preparation and Characterisation

As base composition PbZr_0.6_Ti_0.4_O_3_ was chosen, a rhombohedral ferroelectric perovskite far off the morphotropic phase boundary in the PZT system. For donor-doped samples divalent lead was substituted by trivalent lanthanum with the assumption of ionic compensation by lead vacancies, i.e., two La^3+^ replacing three Pb^2+^. For acceptor-doped samples, tetravalent titanium was substituted by trivalent iron. Ionic compensation would thus lead to oxygen vacancies. In the case of Fe/La co-doping a self-compensation may take place and only the excess of acceptor should be charge-compensated by oxygen vacancies. In all samples the starting material contained 1 mol % of PbO excess to compensate for losses during calcination and sintering. 

The doping concentrations and compositions of all samples are summarised in Table 1. All compositions were prepared by conventional solid state synthesis from commercially available powders of Pb_3_O_4_ (99.99%, Penox GmbH, Köln, Germany), Fe_2_O_3_ (99.99% purity, Merck, Darmstadt, Germany), La_2_O_3_ (min. 99%, Treibacher Industrie AG, Treibach, Austria), ZrO_2_ (Grade 15, MEL Chemicals, Manchester, UK), and TiO_2_ (99.8% purity, Tronox Pigments, Krefeld, Germany). All raw materials were dried at 220 °C and cooled down in a desiccator to avoid moisture effects on weighing. For homogenization the powder mixtures were ball milled in ethanol with tungsten carbide-milling balls 5 mm in diameter in stainless steel beakers lined with a tungsten carbide-inlay using a planetary mill (Pulverisette 7, Fritsch, Idar-Oberstein, Germany). The ball-milling was carried out for 30 min at 300 rpm. After milling, ethanol was removed from the suspensions by keeping them in a drying oven (LUT 6050, Heraeus, Hanau, Germany) at 80 °C over night. The dry mixtures were then sieved through a 500 μm test sieve, transferred into alumina crucibles, covered with lids, and allowed to undergo the solid state reaction at 850 °C for 3 h with a heating rate of 5 K/min in a box furnace (N7/H with C290 controller, Nabertherm, Lilienthal, Germany). 

After the solid state reaction the compounds were again milled in ethanol under the same conditions as before, dried at 80 °C, and sieved to reduce the agglomerate size to less than 180 μm. Before pressing the powder into disc-shaped samples, 5 wt % polyethylene-glycol PEG 20000 (per analysis, Merck, Kenilworth, NJ, USA) was added as a binding agent to ensure sufficient mechanical strength for handling. The samples were pressed with 150 MPa for 5 min into discs of 13 mm diameter and about 1 mm height. To remove the binder before sintering, the samples were heated up to 500 °C in open alumina crucibles to promote the decomposition of PEG to H_2_O and CO_2_. 

Pellets were arranged in a coin roll separated by ZrO_2_ powder. Additionally, atmospheric powder, a 1:1 mixture of Pb_3_O_4_ and ZrO_2_, was put close to the setup to prevent loss of PbO. All was covered with two alumina crucibles. Sintering was performed at 1250 °C for 3 h with a heating rate of 5 K/min in the box furnace (Nabertherm N7/H with C290 controller). After solid state reaction and sintering, the samples were checked by X-ray diffraction (XRD) (Bruker AXS D5005, Cu Kα emitter, graphite secondary monochromator, Bruker, Karlsruhe, Germany). The microstructure of the samples was analysed by a scanning electron microscope (Zeiss Ultra 55, FEG, Carl Zeiss AG, Oberkochen, Germany) in backscattered mode with a working distance of 3–8 mm. This working distance was used in order to perform orientation contrast (OC) imaging. Samples were immersed in epoxy resin, ground with silicon carbide paper, and subsequently polished using 0.25 μm diamond paste. Fine mechanical polishing was performed with 0.04 μm SiO_2_ emulsion for 2 h to obtain a smooth surface and remove any stresses induced during grinding, which would disturb OC imaging. To prevent charging, samples were sputtered with carbon. 

### 2.2. ^18^O Diffusion Experiments and ToF-SIMS Analysis

Information on the oxygen diffusivity of the differently doped PZT samples was obtained by tracer oxygen exchange experiments and subsequent time-of-flight secondary ion mass spectrometry (ToF-SIMS) analysis. The ^18^O_2_ employed for tracer diffusion was purchased from Campro Scientific GmbH (97.1 atm % ^18^O). To avoid significant changes in the ^18^O_2_ partial pressure during the experiment, the tracer amount in the gas phase was very large compared to the oxygen needed for tracer exchange [39]. Prior to evacuation and tracer filling, the samples were pre-annealed for 4 h in ambient air. Tracer diffusion at either 560 °C or 715 °C lasted 30 min. These temperatures turned out to be appropriate in terms of materials stability and depth of the tracer profiles. Since evacuation was performed at annealing temperatures, chemical oxygen diffusion processes could not be avoided, but all measured tracer concentrations were so high that significant effects caused by chemical ^18^O diffusion to equilibrate the oxygen stoichiometry in the sample can safely be neglected. After tracer exchange, the samples were quickly cooled in ^18^O_2_ atmosphere. Any modification of the established tracer profiles by additional tracer diffusion in ferroelectric PZT close to the Curie temperature T_c_ (during quenching) or at room temperature (before SIMS analysis) is believed to be restricted to distances much smaller than the depths of measured profiles. This is due to low diffusion coefficients at room temperatures and small times with exposure to temperatures close to *T*_c_.

To obtain ^18^O depth profiles, a TOF.SIMS 5 instrument (ION-TOF, Münster, Germany) was used with a pulsed bismuth primary ion gun (25 keV, Bi^1+^) working in the “Collimated Burst Alignment” (CBA) mode [40,41]. Applying the CBA mode enables an accurate determination of oxygen isotopic fractions and an improved lateral resolution. The ^18^O fraction fO18 is calculated from the experimentally obtained ^16^O**^−^** and ^18^O**^−^** secondary ion intensities (IO16, IO18) according to fO18=IO18/(IO18+IO16). During analysis the primary ion gun scanned a “field-of-view” of 1024 × 1024 pixel, typically in the range of 50 μm × 50 μm. For depth profiling (up to 1.5 μm depth) a 2 keV Cs^+^ sputter gun rastered an area of 400 μm × 400 μm. The depth of the tracer profiles was calculated by normalizing the sputter times to the sputter crater depths, measured by means of a ZEISS Axio CSM-700 microscope (Carl Zeiss AG, Oberkochen, Germany). In some cases, additional cross-sectional profiles were measured to gain tracer information on a larger length scale. Since PZT is poorly conductive, a 20 nm gold layer was sputtered onto the PZT surface (BAL-TEC MED 020 Coating System, BAL-TEC, Balzers, Liechtenstein) and charge compensation was achieved by an electron flood gun (20 eV).

### 2.3. Impedance Measurements

For electrical characterization, a PZT sample with sputter-deposited Pt electrodes (200 nm) was heated in ambient air to 560 °C, controlled by a thermocouple positioned next to the sample. After temperature stabilization to ±0.5 °C impedance measurements were performed in the range of 10**^−^**^1^–10^6^ Hz using an N4L PSM 1735 (Newton4th, Leicester, UK) in combination with a current amplifier DHPCA-100 (Femto, Berlin, Germany) and 100 mV rms. Most measurements were performed in ambient air. Additionally, different gas compositions were established by using pure O_2_, 1% O_2_ in N_2_ and pure N_2_. Before performing impedance measurements, all virgin samples underwent an equilibration in ambient air until a constant conductivity was reached [27]; this equilibration took place on a time scale of several hours to days and thus probably included also some cation motion. Mechanistic details on the exact processes during this equilibration are not available yet. Conductivity values of differently doped PZT samples are compared after this equilibration.

### 2.4. P–E Measurements

Hysteresis curves were investigated in polarization (*P*)–electrical field (*E*) measurements. Samples were contacted with silver paste (Leitsilber 200, Ögussa, Austria) and *P–E* curves were recorded at room temperature with an aixACCT aixPES system (AixACCT Systems, Aachen, Germany). The electric field was applied with a frequency of 0.1 Hz (triangular shape).

## 3. Results and Discussion

### 3.1. Oxygen Tracer Diffusion in Differently Doped PZT Samples

**Figure 1:**
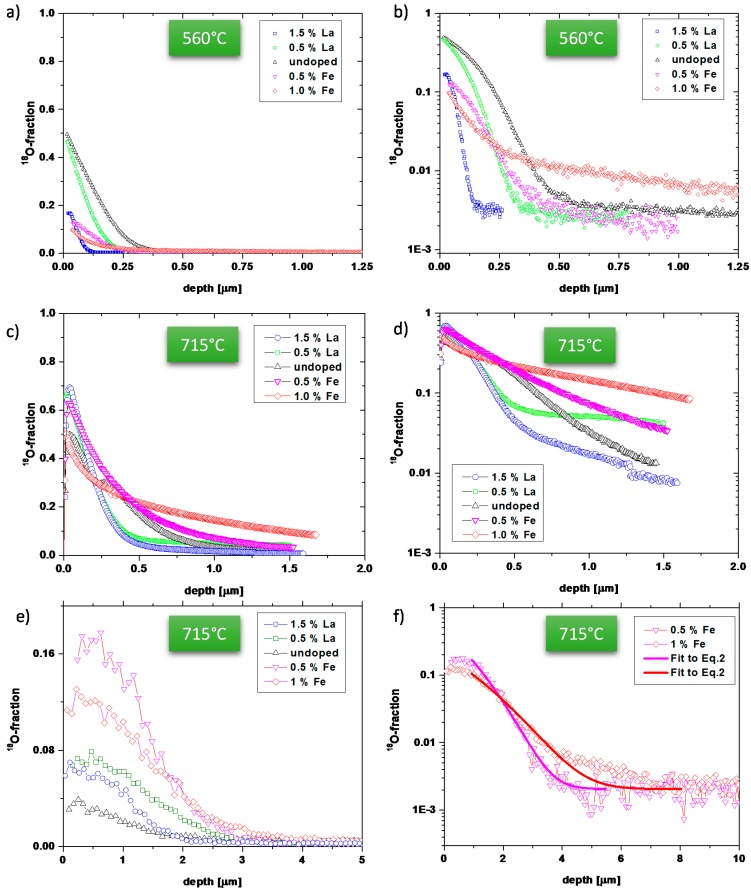
(**a**) Depth profiles of five differently doped lead zirconate titanate (PZT) samples in linear plots after tracer diffusion (30 min) at 560 °C; (**b**) logarithmic plots of the same profiles; (**c**,**d**) depth profiles of tracer exchange experiments at 715 °C (30 min) in linear plots (**c**) and logarithmic plots (**d**); (**e**) cross-sectional measurements after the tracer exchange experiments at 715 °C in linear plots; (**f**) cross-sectional profiles for 715 °C in logarithmic plots with fit curves (Equation (2)) for 0.5% and 1% Fe-doped samples.

**Figure 2:**
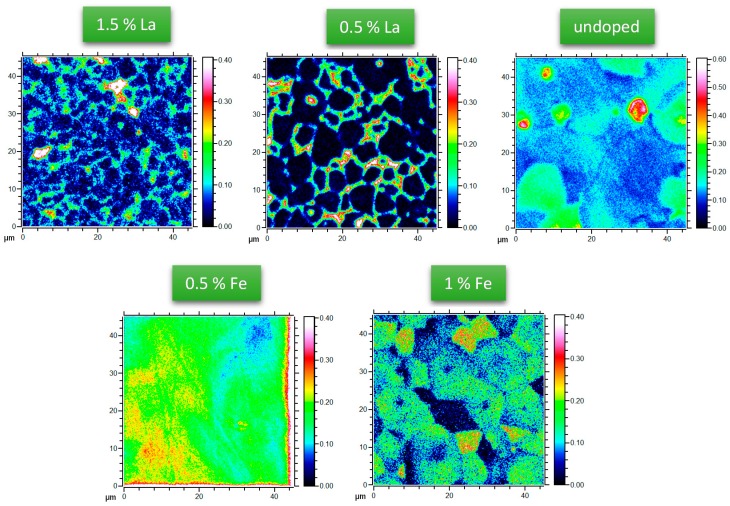
Tracer distribution images of differently doped PZT after diffusion at 715 °C with clear indication of fast grain boundary diffusion in the donor doped samples; ^18^O tracer fractions are shown.

Samples with five different compositions, varying from 1.5% La donor to 1% Fe acceptor doping, were exposed to tracer oxygen for 30 min at 560 °C and 715 °C. The resulting depth profiles were analysed by ToF-SIMS and are shown in Figure 1a–d. For 715 °C cross-sectional profiles, revealing further information on depths > ca. 1 μm, were also measured and are shown in Figure 1e,f. Moreover, in-plane tracer distribution images of the near-surface regions are given in Figure 2 for 715 °C. The depth profiles reveal substantial differences between the samples. 

The highest tracer concentration in several hundred nm depth is found for the high Fe concentration (1%), while the lowest tracer concentration in that depth accounts for the high La concentration (1.5%). This is in accordance with the expectation that donors supress and acceptors enhance the oxygen vacancy concentration and thus the tracer diffusion coefficient. However, quantification of the measured data in terms of diffusion coefficients turns out to be non-trivial, since most depth profiles cannot be described by the simple solution of

(1) ∂fO18∂t=Db*∂2fO18∂x2

for diffusion into a homogeneous half infinite sample from a constant source with limited oxygen exchange (quantified by the oxygen exchange factor k*), i.e., by [39]:

(2) fO18−fObg18fOgas18−fObg18=(1−erf(x2Db*t))−ek*xDb*+k*2tDb*[1−erf(x2Db*t+k*tDb*)]

In Equation (2), fOgas18 denotes the fraction of tracer oxygen in the gas phase and fObg18 the natural abundance of tracer oxygen, *x* = depth, *t* = diffusion time, and $D_{b}^{*}$ = bulk tracer diffusion coefficient.

**Figure 3:**
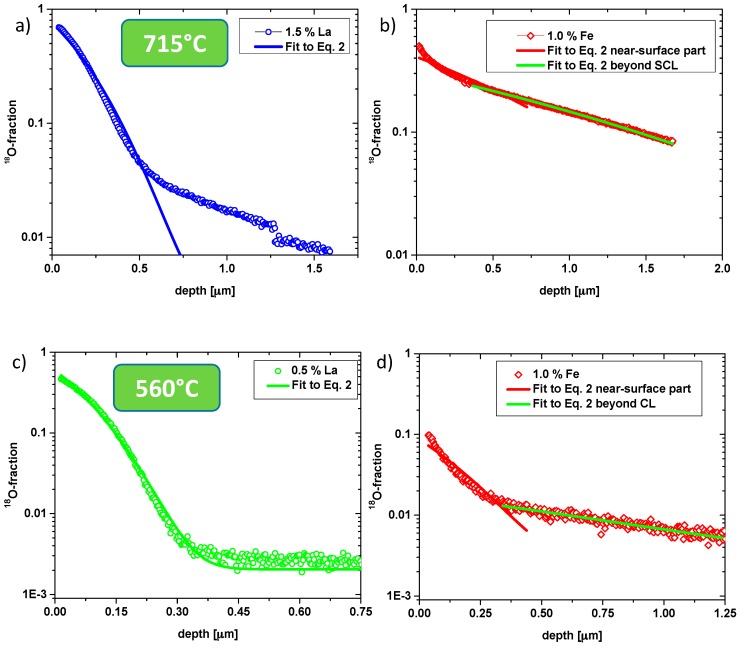
(**a**) Tracer depth profile of 1.5% La-doped PZT at 715 °C with fit according to Equation (2). A clear grain boundary contribution is indicated by the diffusion tail. (**b**) Tracer depth profile of 1% Fe-doped PZT at 715 °C with fits according to Equation (2) in the near surface regime and in the region in some depth (beyond SCL = space charge layer); (**c**) tracer depth profile of 0.5% La-doped PZT at 560 °C with fit according to Equation (2); (**d**) tracer depth profile of 1% Fe-doped PZT at 560 °C with fits according to Equation (2) in the near surface regime and in the region in some depth (beyond SCL).

The discrepancies between experimental data and Equation (2) are exemplified in Figure 3. For La-doped samples, the first part of the profile fits acceptably well to Equation (2) but particularly at 715 °C deviations are very pronounced in some depth. The reason becomes obvious from the tracer distribution images in Figure 2 (715 °C): In La-doped PZT high tracer concentrations are visible at grain boundaries, indicating fast grain boundary diffusion. This is also in accordance with recent publications showing fast grain boundary diffusion in Nd^3+^-doped PZT [42] and Sr/Nb-doped PZT [29,37].

**Figure 4:**
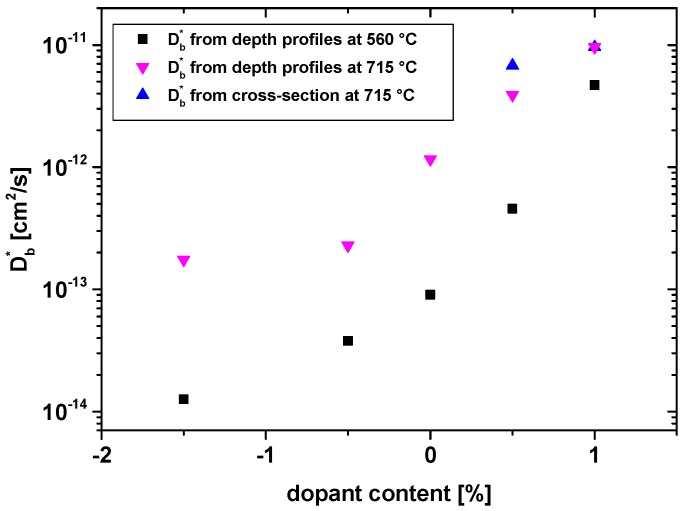
Bulk diffusion coefficients of the five analysed sample compositions. Negative dopant concentrations refer to donor dopant (La), positive to acceptor dopant (Fe).

Hence, the near-surface part of the profiles of La-doped samples was attributed to bulk diffusion and for both temperatures an approximate bulk diffusion coefficient was calculated from a fit of this profile part to Equation (2), see Figure 3a,c. All resulting values are summarized in Figure 4. We may compare the bulk diffusion coefficient of 1.5% La-doped PZT at 560 °C (1.3 × 10**^−^**^14^ cm^2^/s) with the value for Nd^3+^-doped PZT in [42]: There, a bulk diffusion coefficient of ca. 5 × 10**^−^**^14^ cm^2^/s is found at 550 °C, which is in reasonable agreement with our results. Also a tracer diffusion study with higher La concentrations (ca. 8%) leads to very similar diffusion coefficients in the 10**^−^**^14^ cm^2^/s range at 560 °C [36].

Provided that all requirements of Harrison type B diffusion are fulfilled [43], e.g., diffusion length in grains much smaller than the grain size, a grain boundary diffusion coefficient $D_{\mathrm{gb}}^{*}$ can be extracted from the tail part of the profile by using the equation of Whipple [44] and Le Claire [45]:

(3) Dgb*=1.322δ(Db*t)1/2(−∂lnfO18∂x6/5)−5/3

with δ being the grain boundary width. For 715 °C, for example, a grain boundary diffusion coefficient $D_{\mathrm{gb}}^{*}$ = 8 × 10**^−^**^9^ cm^2^/s is found for 0.5% La assuming δ = 2 nm. Hence, grain boundary diffusion is orders of magnitude faster than bulk diffusion in La-doped PZT. However, this is not in the focus of this paper; a detailed discussion of grain boundary diffusion in PZT is given in [27,29,37].

**Figure 5:**
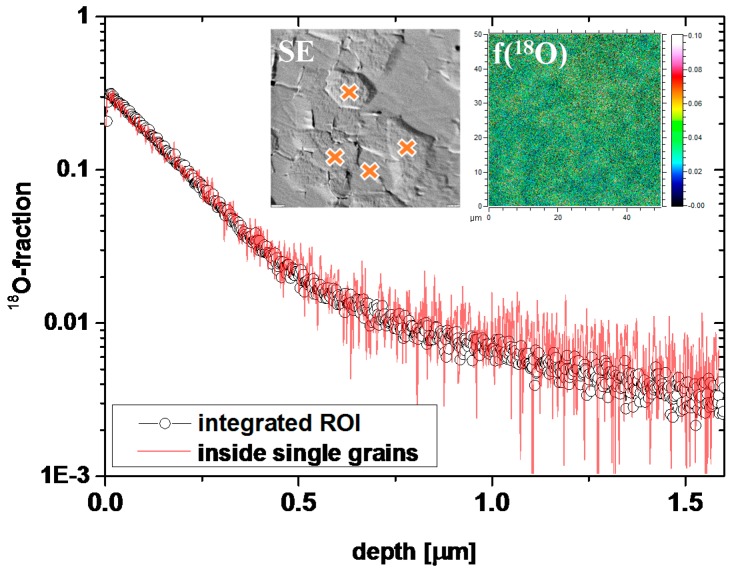
Tracer depth profiles in 1% Fe-doped PZT measured after 30 min tracer diffusion at 560 °C. The circles represent the data after integration over the entire region shown in the tracer distribution image (see insets, *f*(^18^O)). Profiles obtained for small regions of interest (ROIs) within single grains (indicated in the SE inset image) lead to the same profiles.

Also the profiles in Fe-doped PZT could not be well described by Equation (2); severe differences between the shapes expected from Equation (2) and experimental curves are clearly visible in Figure 3b,d for 1% Fe. However, tracer distribution images (Figure 2) do not give any indication of fast grain boundary diffusion and SIMS measurements with a region of interest (ROI) limited to the interior of a single grain showed the same profile as larger sample regions (see Figure 5). Moreover, the near-surface part often showed clear deviations from Equation (2). The much sharper drop of the oxygen tracer fraction in the near-surface region is in agreement with results found for other acceptor-doped perovskite-type oxides (Fe-doped strontium titanate single crystals and undoped PZT films in [38,46,47]). There, this sharp drop was interpreted in terms of a near-surface space charge layer (SCL) with vacancy depletion and thus slow (depth dependent) tracer diffusion. We assume the same for our acceptor-doped samples and thus conclude that in Fe-doped samples the deeper parts of the profiles represent bulk diffusion. Such a space charge with oxygen vacancy depletion can also explain why the near-surface tracer concentration of Fe-doped samples is lower than for the La-doped ones (see Figure 1a,b), despite supposedly more oxygen vacancies in the bulk. 

In accordance with the discussion in [29,48], we included the space charge region into an effective surface exchange coefficient (cf. [27]) and only analysed the bulk-related profile part in some depth by Equation (2) (see Figure 3b,d). This method of analysis may cause some inaccuracy, since setting the limits of the (rather short) bulk type profile part in some depth suffers from a certain arbitrariness. However, cross-section profiles confirmed the validity of the analysis for Fe-doped samples (see below). All values deduced in this manner are plotted in Figure 4. For example, in 0.5% Fe samples a bulk diffusion coefficient of 4.6 × 10**^−^**^13^ cm^2^/s was found at 560 °C.

Undoped PZT did not show clear indication of fast grain boundary diffusion in tracer distribution images but still a kind of tail in the depth profiles at 560 °C that resembles the shape of profiles for donor doping. The first part of the curve fits acceptably well to Equation (2) and hence this part of the profile was attributed to bulk diffusion. At 715 °C, however, such a grain boundary tail cannot be distinguished and the entire profile is used to determine a bulk diffusion coefficient, despite only moderate fit quality. The $D_{b}^{*}$ values for both temperatures are also given in Figure 4. 

The measured cross-sectional profiles (Figure 1e,f) are difficult to quantify, owing to their limited depth and low spatial resolution. For acceptor-doped samples, an analysis of profiles beyond the first μm is possible. In Figure 1f such fits are shown for 0.5% and 1% Fe. The resulting bulk diffusion coefficients are in reasonable agreement with those obtained from the depth profiles (see Figure 4) and this supports the validity of the analysis described above. For donor-doped samples, the near surface bulk part (visible in depth profiles) is not accessible in cross-sectional profiles. Therefore, one might only analyse the grain boundary part of the profile, which was not done in this study. Still, a qualitative comparison of the donor- and acceptor-doped samples is interesting: All effective tracer diffusion lengths visible in Figure 1e are rather similar, even though the mechanisms of tracer transport (bulk diffusion in Fe-doped or grain boundary diffusion in La-doped) are very different. 

Figure 4 summarises all bulk diffusion coefficients determined in the abovementioned ways for 560 and 715 °C. The negative values on the abscissa refer to La donor doping, the zero-point to nominally undoped PZT, and positive values to the Fe-doped samples. These tracer diffusion coefficients are related to the normalized concentration of mobile oxygen vacancies *X*_V_ by [49]:

(4) $$D_{b}^{*}=f_{c}X_{V}D_{V}$$

with *f*_c_ = correlation factor (=0.69 in the perovskite lattice), *D*_V_ = vacancy diffusion coefficient. Trends in vacancy concentrations appear to be reasonable: the much higher tracer diffusion coefficients for acceptor doping are due to much higher oxygen vacancy concentrations. The highest and lowest $D_{b}^{*}$ values are found for the highest acceptor and highest donor concentration, respectively. A quantitative discussion in terms of defect concentrations is given in Section 3.5.

### 3.2. Electrical Conductivity of Differently Doped PZT Samples

**Figure 6:**
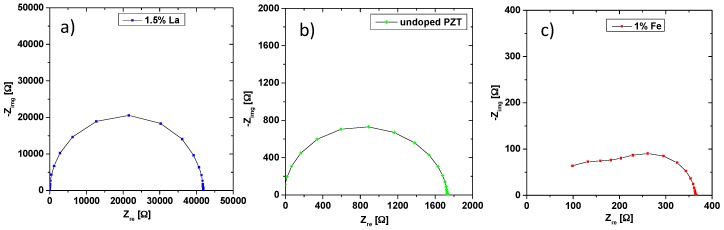
(**a**) Impedance spectrum of the 1.5% La-doped PZT at 560 °C—an almost ideal semicircle results; (**b**) impedance spectrum of the undoped sample at 560 °C; the semicircle is more distorted; (**c**) impedance spectrum of the 1% Fe-doped PZT at 560 °C. A splitting into two semicircles is visible. The absolute resistance values of the three spectra differ by two orders of magnitude.

Electrical conductivity measurements were performed to get insight into the electrically conducting species (electrons or electron holes) and their doping dependence. In Figure 6, the impedance responses of donor-doped (1.5% La), undoped, and acceptor-doped (1% Fe) samples at 560 °C are presented in Nyquist plots. The La-doped samples show an almost ideal semicircle in the complex plane. Fitting of such spectra was done using a parallel connection of a resistor R and a constant phase element (CPE) with impedance:

(5) $$Z_{\mathrm{CPE}}=\;\frac{1}{{\left(i\omega \right)}^{n}\;Q}$$

Fit parameters *n* (~0.98) and *Q* can be used to determine a capacitance [50] and the values fit to the bulk of PZT (relative permittivity of 1576 in the specific case of 1.5% La at 560 °C). This capacitance, together with the absence of any second arc, suggests that the measured conductivity is indeed the bulk conductivity of La-doped PZT, even though a contribution of highly conducting grain boundaries cannot be excluded based on these data [51].

A comparison of the absolute resistance values in Figure 6 shows that Fe-doped samples are much less resistive than the others. Moreover, the impedance response of Fe-doped PZT significantly differs from one semicircle (Figure 6c). Rather, two overlapping arcs are visible and fitting was done to a combination of two serial R-CPE elements. The capacitances of those two semicircles (calculated from *n*, *Q* and *R* [50]) differ by about a factor of five. Similar spectra with two overlapping arcs are found e.g., in [52] for La doped PZT ceramics and there the second arc is attributed to resistive grain boundaries according to the brick layer model [53]. The same interpretation is applied in our case and the only moderate difference of the two capacitances is in qualitative agreement with the assumption that the grain boundary resistance is caused by space charge layers: Due to low bulk carrier concentrations and high permittivities, rather thick space charge layers and thus low grain boundary capacitances may result. 

Space charge zones with thicknesses in the few 100 nm range are also in accordance with the tracer exchange results. There, a space charge depletion layer of similar size was concluded near to the surface, with depletion of oxygen vacancies (see Section 3.1). Undoped PZT shows spectra similar to those of La-doped PZT, even though some asymmetry of the arc is visible in Figure 6b. Still, only a single R-CPE element was used to fit the data and the extracted conductivity is assumed to be that of the bulk.

**Figure 7:**
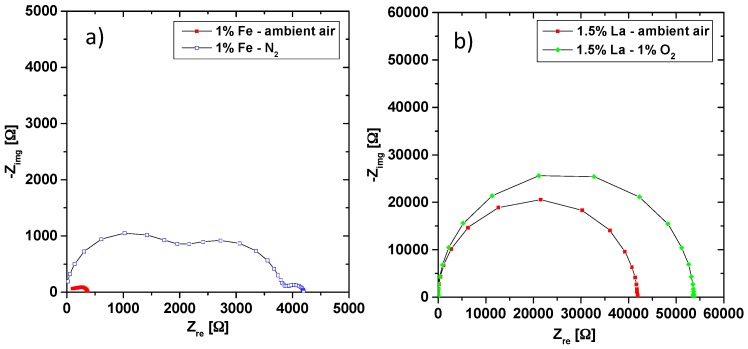
Partial pressure dependence of impedance spectra of 1% Fe-doped (**a**) and 1.5% La-doped (**b**) samples at 560 °C. Both show characteristics of hole conducting material, having a higher resistance for lower oxygen partial pressure.

The partial pressure dependence of the conductivity often gives important information on the type of charge carrier responsible for the electrical current. The oxygen partial pressure dependence of impedance spectra of two different samples is shown in Figure 7. The Fe-doped samples (Figure 7a) show a drastic increase in total resistance when switching from ambient air to nitrogen (typically containing a few ppm O_2_). This increase refers to both the bulk and grain boundary resistance. The occurrence of an additional arc at low frequencies (probably an electrode effect) is not further considered. A detailed defect chemical analysis of the partial pressure dependent defect chemistry of the samples is beyond the scope of this paper, but the increase in bulk resistance with decreasing oxygen partial pressure is a strong indication of predominant hole conduction in these Fe-doped samples. The corresponding defect chemical reaction is

(6) $$O_{O}^{x}+\;{2h}^{•}\;\Leftrightarrow \;1/2O_{2}{+V}_{O}^{••}$$

This hole conduction in acceptor-doped PZT is not surprising and also in accordance with [26,27,31].

After thermal equilibration in ambient air, La-doped PZT was also exposed to a lower oxygen partial pressure (0.01 bar O_2_) and showed an increase in resistance (Figure 7b). The corresponding decrease of conductivity when lowering the oxygen partial pressure was reproducibly measured in all La-doped samples. This is again a strong indication of hole conduction in the material, cf. oxygen equilibration reaction in Equation (6). On the one hand, occurrence of hole conduction in a donor-doped perovskite-type oxide might be surprising. On the other hand, this finding is in accordance with several other studies [26,27,31]. Very similar results are reported in a detailed analysis of Nd^3+^ donor-doped PZT, which revealed hole conduction in a wide partial pressure range [27]. The reason is the high volatility of PbO, which during sintering most probably leads to further cation (lead) vacancies. The surplus of cation vacancies compared to the donor causes a small effective (electron-related) acceptor doping and thus electron holes. 

**Figure 8:**
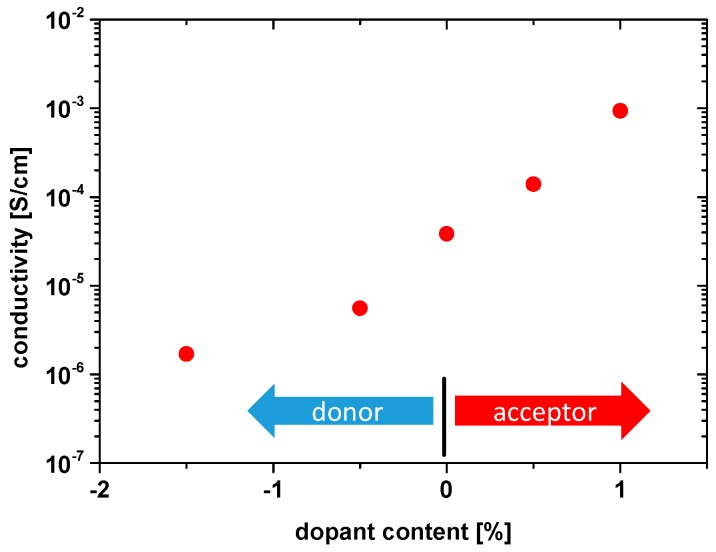
Bulk conductivity values of differently doped PZT at 560 °C in ambient air; negative dopant concentrations refer to donor dopant (La), positive to acceptor dopant (Fe).

For the following comparison, conductivity values were taken from measurements in ambient air after a rather constant conductivity value is reached (donor-doped samples still showed a small variation in time). In Figure 8, the resulting bulk (hole) conductivity values of all samples at 560 °C are shown. The plot resembles that found for tracer diffusion: The hole conductivity is lowest for 1.5% La and highest for 1% Fe. 

### 3.3. Co-Doping of Iron and Lanthanum in Net Acceptor Doped PZT

**Figure 9:**
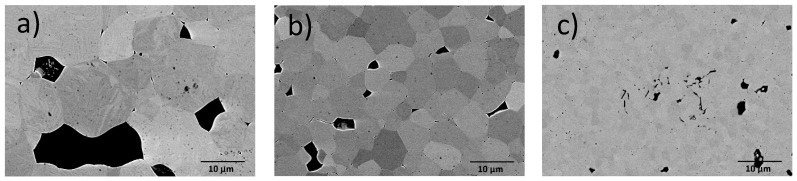
SEM images of co-doped samples, (**a**) 2% Fe/1.5% La; (**b**) 6.5% Fe/6% La; (**c**) 7% Fe/6% La.

In a second sample series, La and Fe dopants were added such that a net acceptor doping of 0.5% or 1% resulted in the ceramic. A net acceptor dopant level of 0.5% was realized by the two pairs (2% Fe/1.5% La), (6.5% Fe/6% La), while an effective acceptor dopant concentration of 1% is expected for (7% Fe/6% La). Figure 9 illustrates the microstructure of the co-doped samples. The grain size decreases with increasing dopant concentration. Only a slight difference is observed for the samples with (2% Fe/1.5% La) and (6.5% Fe/6% La), where the effective acceptor dopant level equals to 0.5%. A marked decrease of grain size is found in the sample with (7% Fe/6% La) with an effective acceptor dopant level of 1%. 

**Figure 10:**
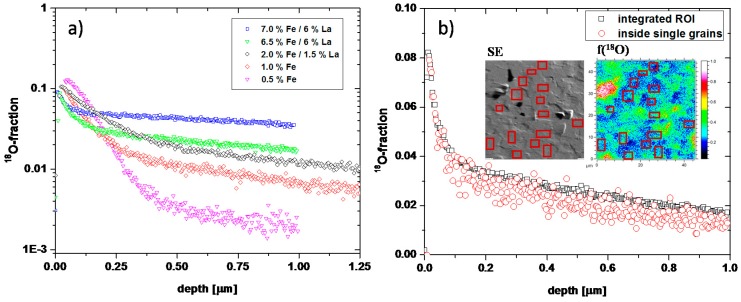
(**a**) Tracer depth profiles after diffusion at 560 °C for all Fe- and Fe/La-doped samples; (**b**) tracer depth profiles of 6.5% Fe/6% La with a region of interest (ROI) integrated over a larger area and ROIs situated within single grains (see SE inset), respectively. The second inset displays the lateral tracer distribution.

Figure 10a shows the tracer depth profiles after oxygen tracer exchange at 560 °C. The shapes resemble those of only Fe-doped samples (also shown in Figure 10): a steeper near-surface decay that cannot be described by Equation (2); and a second part, which can be fitted to Equation (2). Since some of the grains were rather large, profiles within a single grain can again be compared to profiles averaged over larger areas (see Figure 10b). Both profiles are almost identical, supporting the interpretation that fast grain boundary diffusion does not play any role and that the deeper part of the profile again corresponds to bulk diffusion, while near-surface regions are determined by space charge depletion layers. Hence, data analysis was done as described above for Fe-doped PZT.

**Figure 11:**
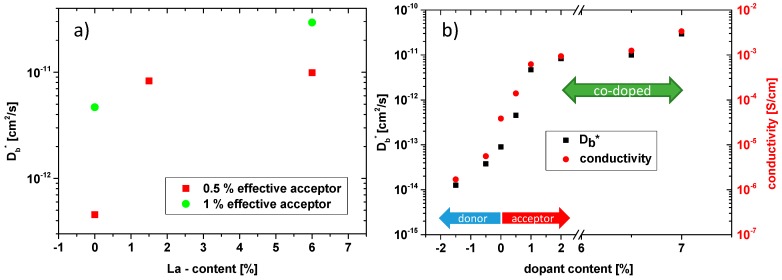
(**a**) Bulk diffusion coefficients of PZT at 560 °C with effective acceptor concentrations of 0.5% and 1%, drawn as function of the La content; (**b**) summary of all bulk diffusion coefficients and hole conductivities in ambient air, measured at 560 °C. For co-doped and acceptor doped samples the abscissa indicates the Fe content.

Figure 11a shows all bulk diffusion coefficients deduced from this analysis for both net acceptor concentrations (0.5% and 1%) as a function of the La content. Despite nominally identical acceptor levels, the diffusion coefficient of the 0.5% series strongly increases when adding La, while the exact amount of La (1.5% or 6.5%) does not play such a role. Also, for nominally 1% acceptor doping, co-doping by La causes an increase in the tracer diffusion coefficient. In both cases the increase of $D_{b}^{*}$ by co-doping is on the order of a factor of 20. We may assume that the oxygen vacancy mobility and thus the vacancy diffusion coefficient *D*_V_ is the same in all our PZT samples. Then the tracer diffusion coefficient is directly related to the concentration of mobile oxygen vacancies by Equation (4). Hence, the concentration of (mobile) oxygen vacancies in all these samples seems to differ much more than one would expect for samples with the entire net acceptor doping being compensated for by oxygen vacancies. This is discussed in more detail in Section 3.5.

**Figure 12:**
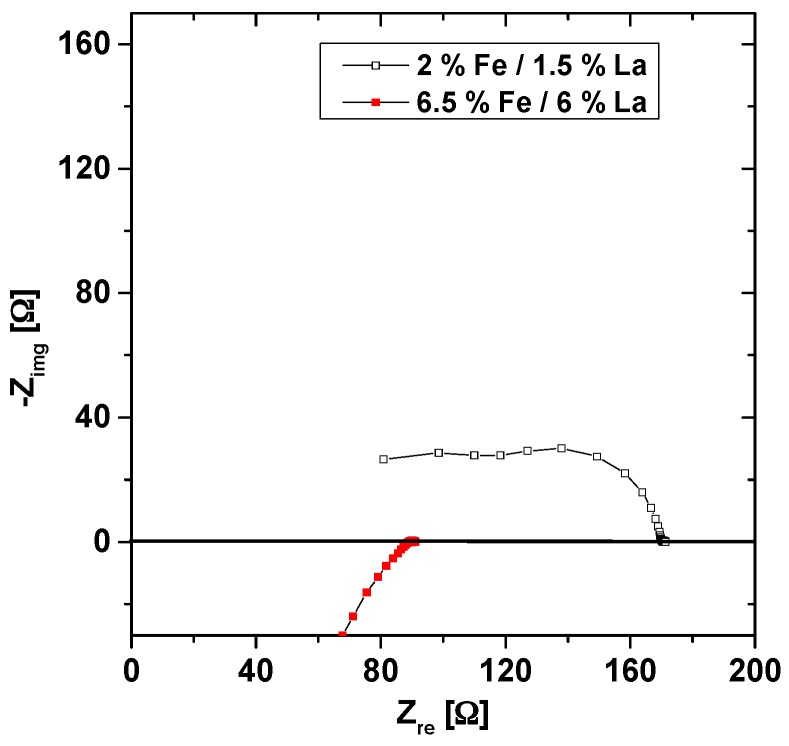
Impedance spectra of PZT with 2% Fe/1.5% La and 6.5% Fe/6% La, measured in ambient air at 560 °C.

Also impedance measurements were performed on co-doped samples at 560 °C (Figure 12) and the spectra show two semicircles, as for purely Fe-doped samples. However, for high co-doping levels, resistances are so small that a reasonable separation of the arcs is no longer possible due to the limited frequency range. For 2% Fe/1.5% La, determination of the bulk conductivity is still possible, while for the higher co-doping only total conductivities including grain boundary resistance could be evaluated from the low frequency intercept. Possibly the grain boundary resistance was absent anyway in these samples, cf. Figure 12. The results are summarized in Figure 11b, together with all other conductivities and bulk diffusion coefficients determined in this study. The trend is obvious: the conductivity of co-doped PZT increases for higher Fe/La concentrations, despite nominally identical net acceptor levels. It is also worth mentioning that for all PZT samples (donor-doped, acceptor-doped, and co-doped) the relative changes of hole conductivity and oxygen bulk diffusion are very similar. The corresponding curves are almost in parallel (Figure 11b) and the difference from the lowest to the highest value is almost identical (a factor of 2000 and 2300 for conductivity and diffusion coefficient, respectively). This is even more interesting since the two properties were obtained by completely different experimental methods. 

### 3.4. P–E Curves

**Figure 13:**
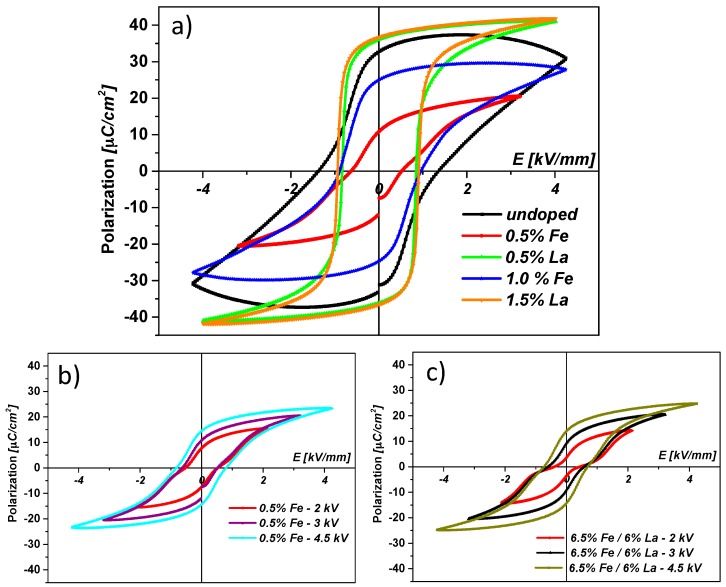
(**a**) *P–E* curves of different PZT samples (see legend). La-doped samples develop slim *P–E* curves with increased polarization. Fe-doped samples develop inclined *P–E* curves with reduced maximum polarization; (**b**) *P–E* curves of a sample doped with 0.5 mol % Fe measured for different maximum electric fields (see legend). Pinching of the *P–E* curve at low field amplitude indicates domain wall pinning, which is typical for acceptor-doped samples; (**c**) *P–E* curves of a sample doped with 6 mol % La and 6.5 mol % Fe measured for different maximum electric fields (see legend). Again the pinching of the *P–E* curves at low field amplitude indicates net acceptor doping of the sample.

*P–E* curves of differently doped PZT samples are shown in Figure 13a. La-doped samples develop slim *P–E* curves with increased saturation polarization compared to undoped or Fe-doped samples. Fe-doped samples, on the other hand, develop an inclined *P–E* curve with reduced saturation polarization compared to undoped PZT. Figure 13b compares the *P–E* curves of samples doped with 0.5% Fe developing at different levels of maximum electric field. The pinching of the *P–E* curves at low field amplitude indicates domain wall pinning, which is typical for acceptor-doped samples. The same feature is observed for samples that are co-doped with 6.5% Fe and 6% La (Figure 13c). This pinching again indicates a net acceptor doping of the co-doped samples. These measurements thus reflect typical hard and soft PZT behaviour for Fe-doped (or co-doped) and La-doped samples, respectively. 

### 3.5. Problems of Interpretation in Terms of a Simple Defect Chemical Model

Qualitatively, the measurements of the first sample series (only one dopant) are in agreement with simple defect chemical models regarding the effect of dopants on oxygen vacancy and hole concentrations: the more acceptors the higher $D_{b}^{*}$ as well as hole conductivity, and the more donors the lower both values are. However, a quantitative analysis reveals that simple defect chemical models cannot explain the results. 

The first remarkable fact deals with the relative difference between measured $D_{b}^{*}$ values at 560 °C. The $D_{b}^{*}$ ratio of 1.5% La and 1% Fe is only about 371; for 715 °C it is even less (54). This difference is small compared to what one might expect for donor- or acceptor-doped perovskites: If all the 1% Fe dopants were in the Fe^3+^ state and charge balanced by oxygen vacancies, we would get a normalized oxygen vacancy concentration *X*_V_ of 1667 ppm with respect to oxygen sites (i.e., 5000 ppm when normalizing to the formula unit). From this value and the measured ratio of bulk diffusion coefficients, we then have to conclude that *X*_V_ = ca. 4 ppm in the 1.5% La-doped sample at 560 °C. This value is rather high for donor-doped perovskites though not impossible, cf. the discussion above on PbO volatility. If, on the other hand, the vacancy concentration in the donor doped samples is much lower, say in the 0.01 ppm range, as suggested in [42] for similar Nd-doped PZT, then Fe doping would lead to many fewer oxygen vacancies than expected (only in the 1 to 10 ppm range, e.g., *X*_V_ = 4 ppm for 1% Fe at 560 °C). 

As a second consideration, let us assume similar oxygen vacancy diffusion coefficients of the two perovskite type materials PZT and SrTiO_3_ (STO). We may take *D*_V_ = 1.1 × 10**^−^**^6^ cm^2^/s at 560 °C for STO from [10] (activation energy = 0.86 eV) and the measured bulk diffusion coefficient in PZT (Figure 4) to calculate vacancy concentrations by Equation (4). This leads to *X*_V_ = 0.005 ppm for 1.5% La and 2 ppm oxygen vacancies for 1% Fe at 560 °C, i.e., much less than required for complete compensation of the acceptor by oxygen vacancies. Complete acceptor compensation by mobile oxygen vacancies would mean a three orders of magnitude lower vacancy diffusion coefficient in PZT compared to STO. This difference would require an activation energy for oxygen vacancy motion in PZT that is 0.5 eV higher than in STO, provided the pre-exponential factors are the same. However, DFT calculations indicate very similar activation energies of oxygen vacancy motion in most perovskite-type oxides [54,55]. Also, a direct comparison of the tracer diffusion coefficients of Fe-doped STO and Fe-doped PZT for similar conditions reveals much larger $D_{b}^{*}$ in STO, despite the existence of significant amounts of Fe^4+^ in STO: 0.3% Fe-doped STO at 700 °C exhibits 3.6 × 10**^−^**^10^ cm^2^/s [48,56,57] compared to 3.9 × 10**^−^**^12^ cm^2^/s at 715 °C for our 0.5% Fe-doped PZT. (Please note that the ferroelectricity of PZT should not play a role here, due to the high temperatures.)

The third unusual effect is the non-linear increase of the tracer diffusion coefficient at 560 °C by one order of magnitude when increasing the Fe concentration from 0.5% to 1% (see Figure 4). This again contradicts the assumption that the Fe-dopant is largely counter-balanced by oxygen vacancies, since in this case only a factor of two should be found. In line with this result is also the strong dependence of the tracer diffusion coefficient on co-doping: despite identical net acceptor concentration, the tracer diffusion coefficient and thus most probably also the oxygen vacancy concentration varies by a factor of about 20 (at 560 °C). This again supports the interpretation that there are generally many fewer oxygen vacancies in PZT than calculated from the upper limit given by complete vacancy compensation of the acceptor dopants in Fe-doped PZT.

Fourth, the conductivity values also reveal discrepancies with a simple defect chemical model: One may assume that mobile holes instead of oxygen vacancies counter-balance the acceptor doping. Then we would have a hole concentration of 0.01 per formula unit or 1.56 × 10^20^ cm**^−^**^3^ (with a cubic lattice constant of 0.4 nm) for 1% Fe-doped PZT. However, if such high concentrations of mobile holes were indeed present, the conductivity should be much larger. Assuming the hole mobility of STO in [10], we would expect more than 1 S/cm, which is several orders of magnitude higher than the measured value. From the mobility of electron holes in Fe-doped STO [10] and the measured conductivity, we might rather estimate the mobile hole concentration in the 1% Fe-doped PZT sample to about 7 ppm per formula unit at 560 °C. Also, the trends in hole conductivities (e.g., an increase by a factor of 6.7 from 0.5% to 1% Fe) contradict the assumption of mobile holes as majority charge carriers that balance acceptors. Moreover, the effect of co-doping on the conductivity cannot be explained on the basis of a simple defect chemical model: the conductivity changes despite identical net acceptor concentration. Finally, the conductivity of hole conducting PZT was often reported to be strongly temperature-dependent, with an activation energy in the 1.1–1.4 eV range [7,58,59,60]. This indicates that a large number of holes is trapped and thus the concentration of mobile holes is strongly temperature-dependent. 

### 3.6. Suggestion of a Modified Defect Chemical Model

Based on all these arguments, we have come up with the hypothesis that neither (mobile) oxygen vacancies nor (mobile) holes are the majority charge carriers that counterbalance the acceptor dopant in PZT. Before detailing this hypothesis, we consider the possibility of explaining the results by vacancy-dopant associates and thus immobile oxygen vacancies. Associations of oxygen vacancies and Fe^3+^ were indeed found in EPR studies [16,17,61] and their properties were modelled in DFT calculations [18,19]. However, the concentration of associated oxygen vacancies should increase with increasing Fe-doping and thus for identical net acceptor doping the fraction of free and thus highly mobile oxygen vacancies should decrease for increasing Fe content in co-doped samples, in contrast to the experimental results for oxygen tracer diffusion. Also, the non-linear increase of $D_{b}^{*}$ with increasing Fe content in Fe-doped PZT disagrees with a simple vacancy association model. Finally, in such a model the concentration of “free” mobile oxygen vacancies is strongly temperature-dependent and thus rather large activation energies of the tracer diffusion coefficient should result. We do not have sufficient data for a reliable analysis of the activation energy, but based on the data given in Figure 4, we expect an activation energy in the 1 eV range or even lower. This is close to the activation energy of oxygen vacancies in perovskite-type lattices [42] and thus does not indicate strong additional temperature-dependent vacancy concentrations. Accordingly, defect associates may be present but they do not solve the problem regarding the charge balancing of acceptors.

Therefore, we suggest trapped holes as the positive majority defect in acceptor-doped PZT. There are several candidates for hole traps in PZT: (i) Cation vacancies may act as hole traps but those have negative relative charges (also with a trapped hole) and thus cannot compensate the charge of any negative acceptor dopant. Rather, they need further positive charge carriers for charge compensation. Hence, cation vacancies, possibly formed during preparation by PbO evaporation, cannot solve our problem regarding the charge balancing. Still, some differences in the PbO loss of our samples during sintering may cause unknown further differences in the net acceptor concentrations and could affect the absolute values of $D_{b}^{*}$ and σ_h_; (ii) Fe^3+^ can trap holes in STO, thus forming Fe^4+^ [10]. Significant Fe^4+^ concentrations may also exist in Fe-doped PZT and we consider such an iron trap as possibly relevant in our PZT samples. However, for several reasons we assume that it is not the only kind of relevant hole trap and possibly also not the most important one: Explaining the 10-fold increase of $D_{b}^{*}$ between 0.5% and 1% Fe by iron traps would require a significant decrease of the trap energy with increasing Fe content. Calculations based on the STO defect chemical data set of [10] also reveal that an increase in the Fe content without changing the net acceptor doping should strongly lower $D_{b}^{*}$, in complete contrast to the experimental data of co-doped samples (see Figure 11b). Fe^3+^ + h^+^ => Fe^4+^ as the only hole-trapping reaction cannot explain the strongly temperature-dependent hole conductivity found in PZT without Fe dopants [42]. Moreover, preliminary measurements with Cu^2+^ doping (0.25%) revealed even lower $D_{b}^{*}$ values than found for analogous iron doping (0.5%), despite the absence of the iron-based hole trap; (iii) We therefore suggest the existence of additional intrinsic neutral traps T^x^ in PZT which become positively charged by holes according to

(7) $$T^{x}+\;h^{•}\;\Leftrightarrow \;{\mathrm{Th}}^{•}\;\mathrm{or}$$

(8) $$T^{x}+\;{2h}^{•}\;\Leftrightarrow \;{T2h}^{••}$$

The existence of Pb^4+^ on A-sites of perovskites is discussed in several publications [62,63,64,65] and such Pb^4+^ ions can be considered as Pb^2+^ with two localized (immobile) trapped holes. Energies of these states are not available so far, but we consider Pb^4+^ as a realistic doubly filled hole trap in PZT. De-trapping in accordance with Equation (8) would then lead to mobile holes. A further possible intrinsic trap is an oxide ion that might form O**^−^** or superoxide ions. Such a hole trap was suggested for TiO_2_ [66] but might be rather shallow. Pb^2+^/Pb^3+^ may also act as a neutral hole trap and the existence of Pb^3+^ is indeed discussed in the literature, but this trap is assumed to be shallow [67,68,69].

In the following we discuss (semi-)quantitatively whether the existence of an intrinsic deep trap could explain some of our observations. More specifically, we assume Pb^4+^ as the positive majority counter-defect of the acceptor dopants. (The same type of calculation, with similar results, can be performed for a singly charged trap.) As a first approximation we quantify the trapping reaction in Equation (8) by its mass action law:

(9) $$K_{T}=\frac{C_{T2h}}{C_{T}\cdot C_{h}^{2}}$$

Symbol *C* denotes concentrations; *C*_h_ is thus the concentration of mobile holes. The total trap concentration *C*_T,tot_ is assumed to be limited and given by

(10) $$C_{T,\mathrm{tot}}=C_{T}+C_{T2h}$$

Introducing this limitation of Pb^2+^/Pb^4+^ trap states is considering that Pb^4+^–Pb^4+^interaction may change the corresponding energy levels and make hole trapping less favourable for high Pb^4+^ concentrations. In a more accurate model, we would have to assume a *C*_T2h_-dependence of *K*_T_ in Equation (9).

The charge neutrality equation for mobile holes (h), oxygen vacancies (V), trapped holes (T2h), and acceptors (A) then reads:

(11) $$C_{A}=2C_{T2h}+2C_{V}+C_{h}$$

In equilibrium with the gas phase, holes and oxygen vacancies are coupled by Equation (6) and with mass action constant *K*_δ_ and oxygen partial pressure *pO*_2_ this leads to

(12) $$\frac{K_{\delta }}{pO_{2}}=K_{\delta }^{\prime }=\frac{C_{V}}{C_{h}^{2}}$$

Combining Equations (9)–(12) results in

(13) $$K_{T}=\frac{\frac{C_{A}}{2}-C_{V}-\frac{1}{2}\sqrt{\frac{C_{V}}{K_{\delta }^{\prime }}}}{\frac{C_{V}}{K_{\delta }^{\prime }}\left(C_{T,\mathrm{tot}}-\frac{C_{A}}{2}+C_{V}+\frac{1}{2}\sqrt{\frac{C_{V}}{K_{\delta }^{\prime }}}\right)}$$

which can be used to calculate the oxygen vacancy concentration for given *K*_T_, $K_{\delta }^{\prime }$, *C*_T,tot_, and *C*_A_.

**Figure 14:**
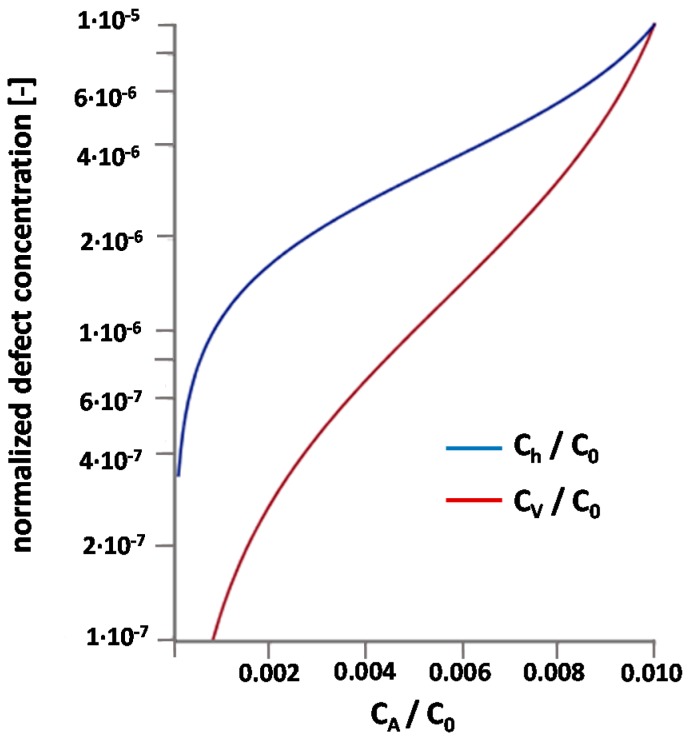
Concentrations of oxygen vacancies and holes for different acceptor concentrations. All concentrations are normalized to a formula unit (concentration of ABO_3_ cell = C_0_) and calculated from Equation (13) with $C_{T,\mathrm{tot}}/C_{0}=0.56\times 10^{-2},K_{T}=0.8\times 10^{11}\times {\left(C_{0}\right)}^{2},K_{\delta }^{\prime }=1\times 10^{5}\times C_{0}$.

Figure 14 exemplarily displays the concentrations normalized to a formula unit for a certain parameter set. A nonlinear increase of *C*_V_ is found for *C*_T,tot_ being not much larger than *C*_A_; the measured increase of *C*_V_ by a factor of 10 at 560 °C can be reproduced in a total Pb^2+^/Pb^4+^ trap concentration of 0.56%. For 1% Fe we then find ca. 10 ppm oxygen vacancies and 10 ppm holes, both with respect to a formula unit (3.3 ppm with respect to oxygen sites). 

Accordingly, this model explains the comparatively low oxygen vacancy concentrations in Fe-doped PZT as well as the strong change of oxygen tracer diffusivity when increasing the Fe content from 0.5% to 1%. Owing to the temperature dependence of $K_{\delta }^{\prime }$ and *K*_T_, the *C*_A_ dependence of *C*_V_ should also be affected by temperature and may become smaller at higher temperatures, in accordance with the experiments. Moreover, the severe effect of co-dopants on defect concentrations, despite identical net acceptor level, is no longer surprising, since the large amount of doping ions might easily change the electronic energy level. For example, if co-doping reduces the Pb^2+^/Pb^4+^ trap energy of one hole (*E*_T_ < 0) by only 80 meV (in $K_{T}=K_{T}^{0}\exp\left(2\left|E_{T}\right|/kT\right)$, the oxygen vacancy concentration increases by an order of magnitude for the dataset in Figure 14. Hence, the assumption of deep hole traps can explain a number of experimental facts that are in contradiction with the simple oxygen vacancy compensation model. 

Finally, we may also consider the strong correlation found between the hole conductivity and the tracer diffusion coefficient (Figure 11b). Qualitatively, the oxygen exchange equilibrium of Equation (6) explains why hole conductivity and tracer diffusion coefficient, i.e., hole concentration and oxygen vacancy concentration, change simultaneously. Equation (12) relates the two concentrations, and for a dopant independent $K_{\delta }^{\prime }$ the vacancy concentration is expected to change even more than the hole concentration ($C_{V}\propto C_{h}^{2}$). Table 2 summarizes all relative changes of hole conductivity σ_h_ and oxygen bulk diffusion coefficient $D_{b}^{*}$ for the first measurement series. Ideally, ${\left(\sigma_{h}\;\mathrm{ratio}\right)}^{2}/\left(D_{b}^{*}\;\mathrm{ratio}\right)$ (last column in Table 2) should be unity. However, in all cases the hole conductivity changes more than expected (or the tracer diffusion coefficient less than expected) and hence an additional deviation from a simple defect chemical situation seems to be present in PZT, e.g., $K_{\delta }^{\prime }$ may depend on the dopant concentration due to defect interactions. 

Based on the reasonable agreement of many experimental facts with the model of deep hole traps in PZT, we consider it a realistic hypothesis that acceptor-doped PZT has many fewer oxygen vacancies than one might expect from the dopant level. In our case we suggest only a few ppm oxygen vacancies for 1% Fe. Possibly, acceptors are largely charge-balanced by trapped holes. Presumably the trapped holes are not only present as Fe^4+^ but also populate an intrinsic hole trap such as Pb^2+^/Pb^4+^. Donor-doped PZT, on the other hand, has a measureable bulk vacancy concentration, probably in the sub-ppm range (0.01–0.1 ppm in our case), and a substantial oxygen vacancy concentration in grain boundaries. The latter is discussed in more detail in [32,42]. 

These results might also affect the discussion of PZT hardening by acceptor doping, since in the current models of hardening oxygen vacancies play a key role (see e.g., [18,19,20,22,23]). It is an open question whether oxygen vacancies have a large effect on domain motion even if their concentration in acceptor-doped PZT were low (in the several ppm range). Moreover, the interaction of domain walls with the supposed trapped holes might affect the ferroelectric properties of PZT. Future experimental and theoretical work may prove or disprove our hypothesis of deep hole traps. Experimentally, for example, additional oxygen partial pressure dependent measurements of conductivity and tracer diffusion may help elucidate the defect chemistry of PZT.

## 4. Conclusions

Oxygen tracer diffusion in donor (La^3+^)- and acceptor (Fe^3+^)-doped PZT revealed different predominant diffusion mechanisms. In donor-doped PZT grain boundary diffusion prevails, with little though still measureable oxygen bulk diffusion. Acceptor-doped PZT shows higher bulk diffusion coefficients without any indication of fast grain boundary diffusion. The different bulk diffusion coefficients of donor- and acceptor-doped PZT reflect the varying oxygen vacancy concentrations in the two materials; differences are up to about a factor of 400 for the 1.5% La and 1% Fe concentrations used here. However, variations are less pronounced than one might expect for this severe dopant change, and in particular differences are less than one could expect from an almost complete oxygen vacancy compensation of the acceptor dopants. Oxygen vacancy concentrations of only several ppm are estimated for our Fe-doped PZT. Conductivity measurements revealed hole conduction in both doping cases, with higher conductivity values in acceptor-doped PZT. However, the conductivity is much lower than supposed for a substantial compensation of acceptor doping by mobile holes. Co-doping by acceptors and donors changes the diffusion coefficients and conductivities even when keeping the net acceptor dopant concentration constant. 

The combination of all these observations cannot be simply explained by common defect chemical models, e.g., by the assumption of a predominant oxygen vacancy compensation of the net acceptor doping. Instead, we suggest that neither oxygen vacancies nor mobile holes, but trapped holes are the majority defect counterbalancing the acceptor doping in PZT. Besides Fe^4+^, intrinsic hole traps such as Pb^4+^ ions on A sites may play an important role. This hypothesis of acceptor compensation by trapped holes might also affect the further discussion of processes leading to the hardening of PZT. 

## Figures and Tables

**Table 1:** Summary of lead zirconate titanate (PZT) samples investigated in this study. The amounts of dopant are given in mol % according to the perovskite formula. In the formula of the composition ionic compensation of donors is taken into account by cation vacancies.

Dopants	Net Acceptor Doping (Singly Charged)	Composition
Undoped	0%	PbZr_0.60_Ti_0.40_O_3_
0.5% La	-	Pb_0.9925_La_0.005_Zr_0.6_Ti_0.4_O_3_
1.5% La	-	Pb_0.9775_La_0.015_Zr_0.6_Ti_0.4_O_3_
0.5% Fe	0.5%	PbZr_0.60_Ti_0.395_Fe_0.005_O_3_
1% Fe	1%	PbZr_0.60_Ti_0.39_Fe_0.01_O_3_
2% Fe, 1.5% La	0.5%	Pb_0.985_La_0.015_Zr_0.60_Ti_0.38_Fe_0.02_O_3_
6.5% Fe, 6% La	0.5%	Pb_0.94_La_0.06_Zr_0.60_Ti_0.335_Fe_0.065_O_3_
7% Fe, 6% La	1%	Pb_0.94_La_0.06_Zr_0.60_Ti_0.33_Fe_0.07_O_3_

**Table 2:** Ratios of conductivities and tracer bulk diffusion coefficients at 560 °C.

Samples	$\sigma_{h}\;\mathrm{Ratio}$	$D_{b}^{*}\;\mathrm{Ratio}$	$\frac{{\left(\sigma_{h}\;\mathrm{Ratio}\right)}^{2}}{\left(D_{b}^{*}\;\mathrm{Ratio}\right)}$
0.5% La/1.5% La	3.3	3.0	3.63
undoped/0.5% La	6.3	2.4	16.5
0.5% Fe/undoped	4.0	5.1	3.2
1% Fe/0.5% Fe	6.7	10.2	4.4
